# The Impact of Gestational Diabetes Mellitus (GDM) on the Development and Composition of the Neonatal Gut Microbiota: A Systematic Review

**DOI:** 10.3390/microorganisms12081564

**Published:** 2024-07-31

**Authors:** Rozeta Sokou, Eirini Moschari, Alexia Eleftheria Palioura, Aikaterini-Pothiti Palioura, Alexandra Mpakosi, Theodoula Adamakidou, Eugenia Vlachou, Martha Theodoraki, Nicoletta Iacovidou, Athanasios N. Tsartsalis

**Affiliations:** 1 Neonatal Intensive Care Unit, General Hospital of Nikea “Agios Panteleimon”, 18454 Piraeus, Greece; eirini.moshari@gmail.com (E.M.); al.palioura@gmail.com (A.E.P.); kpalioura@gmail.com (A.-P.P.); anastasiosmmr@yahoo.gr (M.T.); 2 Neonatal Department, National and Kapodistrian University of Athens, Aretaieio Hospital, 11528 Athens, Greece; niciac58@gmail.com; 3 Department of Microbiology, General Hospital of Nikea “Agios Panteleimon”, 18454 Piraeus, Greece; alexiabakossi@yahoo.gr; 4 Department of Nursing, School of Health Sciences, University of West Attica, Ag. Spydironos 28, 12243 Athens, Greece; thadam@uniwa.gr (T.A.); evlachou@uniwa.gr (E.V.); 5 Department of Endocrinology Diabetes and Metabolism, Naval Hospital of Athens, Dinokratous 70, 11521 Athens, Greece; atsartsalis@uniwa.gr

**Keywords:** gestational diabetes, microbiota, neonatal morbidity, neonatal mortality

## Abstract

Gestational diabetes mellitus (GDM) is an important health issue, as it is connected with adverse effects to the mother as well as the fetus. A factor of essence for the pathology of this disorder is the gut microbiota, which seems to have an impact on the development and course of GDM. The role of the gut microbiota on maternal reproductive health and all the changes that happen during pregnancy as well as during the neonatal period is of high interest. The correct establishment and maturation of the gut microbiota is of high importance for the development of basic biological systems. The aim of this study is to provide a systematic review of the literature on the effect of GDM on the gut microbiota of neonates, as well as possible links to morbidity and mortality of neonates born to mothers with GDM. Systematic research took place in databases including PubMed and Scopus until June 2024. Data that involved demographics, methodology, and changes to the microbiota were derived and divided based on patients with exposure to or with GDM. The research conducted on online databases revealed 316 studies, of which only 16 met all the criteria and were included in this review. Research from the studies showed great heterogeneity and varying findings at the level of changes in α and β diversity and enrichment or depletion in phylum, gene, species, and operational taxonomic units in the neonatal gut microbiota of infants born to mothers with GDM. The ways in which the microbiota of neonates and infants are altered due to GDM remain largely unclear and require further investigation. Future studies are needed to explore and clarify these mechanisms.

## 1. Introduction

Gestation is a complicated process that is influenced by a variety of interrelated molecular and cellular mechanisms [1]. During pregnancy, many functional, hormonal, immunological, microbiological, and metabolic changes take place, which aim to maintain the homeostasis of the body and, at the same time, cover the increased needs of the fetus [2,3]. Disorders that may occur during pregnancy can have immediate effects on both the mother and the neonate, and these effects can extend into long-term health complications for both. One such change is a temporary state of hyperglycemia. As pregnancy progresses, particularly during the third trimester, there is an increase in pro-inflammatory cytokines and metabolic hormones, which causes a reduction in insulin sensitivity. In some cases, this can lead to the development of a pathological condition known as gestational diabetes mellitus (GDM), characterized by glucose intolerance during pregnancy [4,5].

GDM is a great problem of public health, given the repercussions on the mother and the fetus, as well as its potential long-term effects on overall health. The prevalence of GDM has increased throughout the years and affects an important number of pregnancies [6]. GDM is classified as the ninth cause of death when it comes to mortality rates worldwide. In 2021, 537 million people were diagnosed with diabetes, 231.9 million people were undiagnosed, and there were 6.7 million deaths worldwide [7]. In the case of diabetes during pregnancy, the worldwide predominance was 21.1 million in 2021, of which 80.3% was GDM and 9.1% were other types of diabetes that were diagnosed for the first time during pregnancy, whereas 10.6% had been traced before pregnancy [7]. The prevalence of GDM differs according to nationality and socioeconomic state. The lowest prevalence has been found in non-Hispanic women (4.2%).

GDM has been correlated with various consequences for the mother and neonate—for example, the need for caesarian section, preeclampsia, preterm birth, large-for-gestational-age (LGA) neonates, shoulder dystocia, hypoglycemia, and other complications for the neonate. Women diagnosed with GDM and their infants are at an increased risk of developing type 2 diabetes, obesity, heart disease, and other metabolic disorders in the future.

According to Hyperglycemia and Adverse Pregnancy Outcome (HAPO) reports, hyperglycemia in the mother is a critical factor that causes multiple complications for the mother and fetus [8]. Other emerging data show that these children may be at a greater risk of developing atopic dermatitis and sensitivity to allergens. One clinical trial noted that infants born to mothers with GDM were more sensitive to allergens and that the risk of sensitivity increases more than five times. It is also more likely that they will suffer from atopic dermatitis, which increases the risk of sensitivity up to seven times [9]. There an increasing interest to the study of the role of the gut microbiota in maternal reproductive health and the changes that take place during the gestational and neonatal period.

The microbial population that colonizes humans, collectively known as the human microbiota, forms a complex ecosystem uniquely adapted to the physiological fluctuations of its host [10]. It is estimated that the human organism is colonized by 10^14^ microbial cells, which cohabitate in different parts of the human body, with the majority found in the gut. The microbial flora is a collection of microorganisms that reside in mucous surfaces and the skin in a coexisting relationship. The gut, for example, contains an estimated number of 100 trillion bacteria, archeobacteria, viruses, and eukaryotic microorganisms that mainly colonize the peripheral colon [11]. The microbial flora of the human genital tract, mouth, respiratory system, and uterus are also main substances in mucous surfaces [12].

The human microbiota is considered to contribute to various physiological and pathological mechanisms. Immune regulation of the host; defense against pathogenic bacteria by preventing their attachment to mucosal surfaces; and the digestion, metabolism, production, and extraction of nutrients and vitamins absorbed by epithelial cells are some of the proposed functions of the human gut microbiota [12,13]. Studies have found a link between the human microbiota and various pathologies such as metabolic syndrome and its components, including obesity, hyperglycemia, and insulin resistance [14,15]. Various interpretations have been suggested behind these pathologies. The microorganisms of the gut microbiota contribute to the production of metabolites like small-chain fatty acids (SCFA), for example, butyric and propionic acid, which play an important role in the preservation of the integrity of the enteric barrier, inhibiting the escape of pathogenic and toxic bacterial products such as lipopolysaccharides in blood circulation [16]. It is believed that microbial flora causes obesity through the fermentation of dietary fibers, inducing an overproduction of SCFAs. Moreover, it can lead to the development of type 2 diabetes by increasing transportation through the membrane of sugars and branched-chain amino acids and also augmenting the response to oxidative stress [17]. Furthermore, it is believed that a defective microflora induces a resistance to insulin through a small grade of inflammation that takes place through lipopolysaccharide (LPS) pathways that are fairly increased in patients that consume a diet high in fat [13,18].

The sum of microorganisms that grow in various human cavities comprise the human microbiota, while the “microbiome” encompasses the collective genetic material of the microbiota, as well as the interactions between these microorganisms and their environment [19]. In total, ¾ of a person’s microbiota can be inherited by the mother when the neonate is exposed to the microorganisms of the genital pathway during childbirth [20]. Moreover, the mother’s microbiota from the mouth, feces, skin and placenta may contribute to primary microbiological habitation and growth of the neonate’s microbiota. The mother’s milk plays an important role in the maturity and profile of the neonate’s microbiota after birth [21]. Disturbance of the balance between normal bacterial populations of the microbiota (known as dysbiosis) could make an impact on the mother’s metabolic profile and contribute to gestational complications, therefore having an influence on the fetus’s health [22,23]. The appropriate establishment and maturation of the neonatal gut microbiota is necessary for the production of various basic biological systems.

The gut microbiota, which is known as the “second brain”, consists of bacteria that play an important role in various natural processes [24,25]. Studies have shown that it plays an important role in digestion, in the immune system, in neurological transmission, in hormone regulation, in the metabolism of medications and toxins, and in the production of metabolites that affect the physiology of the host [26]. The mother’s health has a great impact on the neonate’s gut microbiota. The primary distortion of the gut microbiota has been linked to inflammatory, allergic, and other metabolic disorders of the immune system later on in life [27]. Lately, special interest has been given to the study of a link between the microbial species in a woman’s body and the development of GDM. It has been noticed that the microbiota undergoes certain changes during pregnancy as well as after childbirth, especially in the gastrointestinal tract, the mouth, and the genital tract [28,29]. The latest studies show that the microbiota plays an important role in the development and management of GDM. However, the mechanisms through which the metabolism is affected and how the microbiota can be altered so that the health of women with GDM can be improved still remain unknown. Additionally, given the location-specific variation in physical and chemical environments in the gastrointestinal tract, it is reasonable to assume that the gut microbiome composition might vary depending on the specific location. Indeed, research has shown that the fecal microbiome has a limited capacity to represent the entire microbiome of the host’s gut. The fecal microbiome is simply a subset of the gut microbiome and not fully representative of the host’s gastrointestinal tract. This limitation hinders the identification of causative intestinal microbes associated with phenotypes and diseases when relying solely on fecal microbiome studies [30]. Furthermore, the consequences these changes in the mother’s microbiota have on the gut microbiota of the neonate are still under study [31].

This study aims to systematically review the literature on the impact of GDM on the gut microbiota of neonates. Additionally, it seeks to explore the potential link between gut microbiota and the mortality and morbidity of neonates born to mothers with GDM.

## 2. Materials and Methods

This study employs a systematic review methodology to identify, evaluate, and interpret available studies related to the research objective. For this systematic review, a protocol was developed following the guidelines of the Preferred Reporting Items for Systematic Reviews and Meta-Analyses (PRISMA, PRISMA checklist was presented as a Supplementary Materials) [32], which has been registered in the PROSPERO database (CRD42024556383; https://www.crd.york.ac.uk/prospero/export_details_pdf.php; accessed on 9 July 2024).

### 2.1. Inclusion Criteria

Randomized clinical trials (RCTs), cohort studies (prospective or retrospective), observational studies (regardless of the number of patients included or the origin center), case–control studies, and cross-sectional studies that aim to evaluate the composition of the gut microbiota of neonates born to mothers with GDM were included. Only studies written in English were considered, without geographical or chronological restrictions.

The PICO (Population, Intervention, Comparator, Outcome) study design was used to form the basic search terms during the process of selecting references and choosing studies, as well as to define eligibility criteria.

### 2.2. Research Question, Focused According to PICO

Population (P): neonates born to mothers with GDM;

Intervention (I): exposure to intrauterine conditions of diabetes;

Comparator (C): neonates that were not exposed to intrauterine conditions of diabetes;

Outcome (O): the composition of the gut microbiota.

### 2.3. This Study’s Exclusion Criteria

- Studies that included neonates of mothers with GDM but did not assess the composition or biodiversity of the infant’s gut microbiota beyond defining the abundance of a specific species only;
- Studies concerning the neonate’s gut microbiota that did not exclusively focus on neonates of mothers with GDM;
- Studies from conference proceedings limited to abstracts;
- Case series, case reports, book chapters, guidelines, letters to the publisher, studies concerning systematic reviews of the literature and meta-analyses;
- Studies that were not published in the English language;
- Studies concerning experimental animal models.

### 2.4. Studied Outcomes

The initial outcomes that were evaluated were (1) the composition of the gut microbiota of neonates born to mothers with GDM and (2) the assessment of potential changes in the gut microbiota of these neonates compared to the gut microbiota of neonates not exposed to intrauterine diabetic conditions.

The secondary outcomes assessed were the potential correlation between the gut microbiota and the morbidity and mortality of neonates of mothers with GDM.

### 2.5. Search Strategy—Databases

A systematic review of the literature was conducted from May 2024, with the final date set at the 9 June 2024. The systematic review of the existing literature was conducted using searches in the electronic databases PubMed and Scopus.

The combination of key-words used was “infant”, “neonate”, “premature”, “preterm”, “preterm infant”, “premature infant”, “preterm neonate”, “premature neonate”, “microbiome”, “microbiota”, “flora”, “gut microbiome”, “gut microbiota”, “gut microbes”, “gut flora”, “gastrointestinal microbiome”, “gastrointestinal microbes”, “gastrointestinal microbiota”, “gastrointestinal flora”, “intestinal microbiome”, “intestinal microbiota”, “intestinal microbes”, “intestinal flora”, “fecal microbiome”, “fecal microbiota”, “fecal microbes”, “gestational diabetes”, “gestational diabetes mellitus”, “GDM”, “pregnancy induced diabetes”, “gestational hyperglycemia”, “gestational glucose intolerance”, “type 1 diabetes mellitus and pregnancy”, “type 1 diabetes and pregnancy” or “diabetes mellitus type 1 in pregnancy” or “diabetes type 1 in pregnancy” and “type 2 diabetes in pregnancy” or “type 2 diabetes mellitus in pregnancy” and “pre-GDM”, “diabetes in pregnancy” with Boolean logical operators (AND, OR). Additionally, in order to limit the risk of missing studies and cover the entire scope of the available literature, an investigation and review of the bibliographic references of each selected study were conducted, as well as references from previous systematic reviews within the same research field.

### 2.6. Conflict Resolution

The review, data extraction, and quality assessment were conducted independently by two researchers (M.T., E.M.), with conflicts resolved through discussion and consensus between them or, if necessary, by a third researcher (R.S.).

### 2.7. Composition and Data Presentation

We recorded the data in table format, categorized by main author, study design, date of publication (regardless of whether there was chronological ambiguity or not), country in which the study took place, the neonate’s day of life during sample collection for examination, type of sample, technique used and how the microbiota was analyzed, criteria that were used to define the presence of GDM in mothers, the increase or decrease in the microbial profile (phylum, class, family, genus) in comparison to the healthy control group, number of participants, and number and other relative grouping criteria of the study population (subpopulations of mothers depending on whether they had pre-existing diabetes before pregnancy or GDM managed by diet or insulin; subpopulations of newborns such as preterm infants, very-low-birth-weight infants), with the aim of gathering and meta-analyzing the study results, if possible. We described potential gaps in our evidence and provided suggestions for future research.

## 3. Results

**Figure 1:**
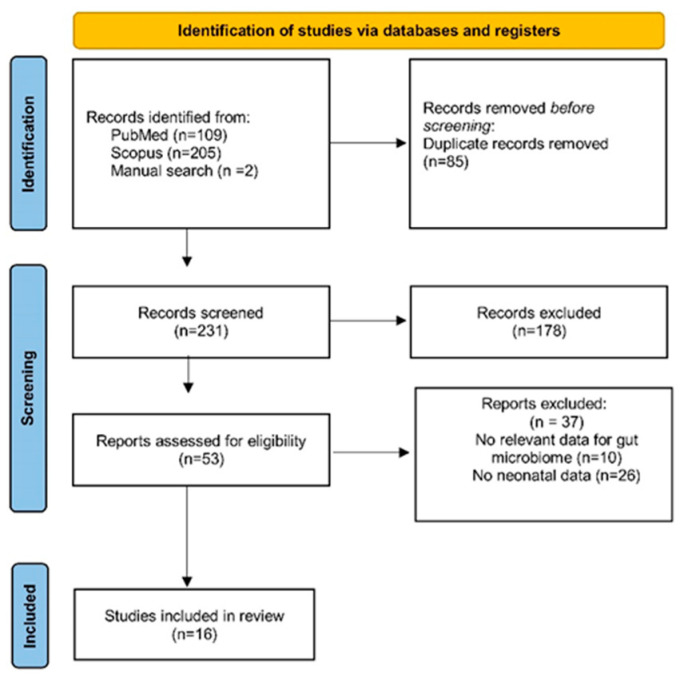
Systematic review flowchart.

A total of 316 studies were retrieved from the search in electronic bibliographic databases. From this total, 85 were duplicates and were removed. The removal of duplicates was performed with the duplicate removal tool of the bibliographic reference management program (EndNote X8). After carefully reading the titles and abstracts of the 231 remaining studies, 178 studies were excluded either because their subject matter did not serve the purpose of this study or because they met some of the exclusion criteria, already apparent in the title or the abstract. A careful reading of the full text of the remaining 53 studies revealed that only 16 studies met all the inclusion criteria and were included in this review [1,2,6,33,34,35,36,37,38,39,40,41,42,43,44,45]. A flowchart of this review process is presented in Figure 1.

### 3.1. Characteristics of the Studies Included in the Systematic Review

All of the studies that were included in the systematic review were observational studies. The characteristics of each study are presented in detail in Table 1.

**Figure 2:**
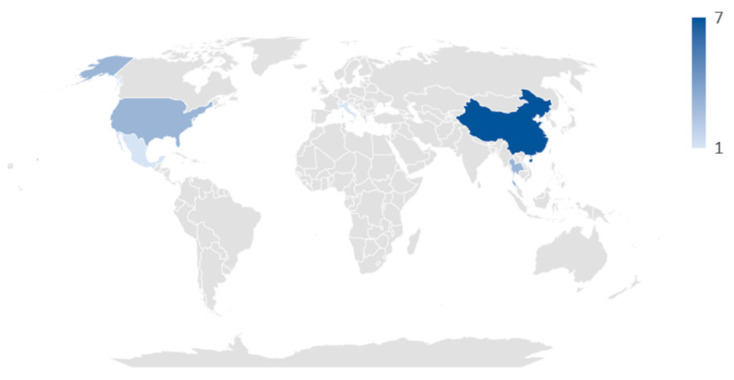
Map of the countries of origin of the studies included in the systematic review.

In total, these studies evaluated data concerning the analysis of the gut microbiota from 1485 neonates, out of which 578 (38.9%) were neonates with GDM, 745 (50.2%) were neonates of mothers without GDM, and for the remaining 162 (10.9%), information was not available. The countries from where the studies originate are Thailand [35,38], China [1,6,36,40,41,42,44], Denmark [2], USA [33,39], Italy [37,45], and Mexico [43] (Figure 2).

The timing of the microbiome analysis of the neonates of mothers with or without GDM varied between studies, with a time frame ranging from the first hours of life to 4 weeks. In seven studies, it is mentioned that successive analyses of the microbiota were performed in different time periods [2,35,36,38,40,43,45]. One study assessed the offspring’s microbiota up to the age of 4 years old [45]. Regarding the diagnosis of GDM, various criteria were used, such as Chinese diagnostic criteria, the Guidelines for the Prevention and Treatment of Type 2 Diabetes in China, international criteria, the criteria of the World Health Organization (WHO), hospital diagnostic criteria, the criteria of the National Diabetes Data Group (NDDG), the Carpenter and Coustan criteria, and the criteria of the International Association of Diabetes and Pregnancy Study Groups (IADPSG). The inclusion and exclusion criteria of women and their offspring—however, not identical—were mostly similar between the groups concerning medical history and medication. In many of the studies that were included in the review, women were excluded if they had used antibiotics in the last 2 weeks to 3 months before delivery, if they had a medical history of chronic diseases or comorbidities such as hypertension, renal, hepatic, and gastrointestinal diseases and infections, and if they had consumed probiotics before entering the study. Regarding pre-existing diabetes mellitus, it was clearly stated as an exclusion criterion in only seven studies [1,6,34,36,37,39,40], while there is one study in which the effect of pre-existing type 2 diabetes and GDM on the neonatal gut microbiome was evaluated [35]. All of the studies used the 16S rRNA sequencing method in order to define the synthesis of the microbiome.

### 3.2. Gut Microbiome in the Offspring of Women with GDM

Most of the studies that were included in the systematic review present important changes in the gut microbiome in the offspring of women with GDM, including a reduction in α- and β-diversity and changes in the relative abundance of certain specific bacteria. The gut microbiota of neonates born to mothers with GDM often exhibited differences compared to that of neonates born to healthy mothers. Specifically, changes in the composition and diversity of intestinal microorganisms were observed, including increased abundance of pro-inflammatory bacteria and reduced α- and β-diversity. These changes can impact the health of neonates, increasing the risk for various metabolic problems in the future. Traditionally, *Bacteroidetes* and *Firmicutes* represent the majority of the gut microbiota, as primarily confirmed by the relative abundance data collected in the analyzed studies (Table 2). 

Furthermore, the *Firmicutes/Bacteroidetes* (F/B) ratio is often used as a dysbiosis index. However, due to the vast heterogeneity of the studies, meta-analysis was not possible, in order to highlight or exclude any statistically significant differences in microbiome composition between neonates born to mothers with GDM and the control group. Chen et al. [1], analyzing 418 meconium samples from neonates born to 147 women with GDM and 271 women who had normal pregnancies, using 16S rRNA gene sequencing, concluded that the microbial communities in meconium were significantly altered in neonates born to mothers with GDM. In terms of phylum, the abundance of *Firmicutes* and *Proteobacteria*, significantly changed in neonates born to mothers with GDM. There was a significant reduction in α-diversity (Chao1 index: *p* < 0.001) in neonates born to mothers with GDM compared to those born to mothers without GDM. Additionally, GDM was significantly associated with changes in β-diversity. B-diversity was significantly different according to type of delivery. Interestingly, a similar trend of change in bacterial families was observed in different modes of delivery, revealing the coherence of microbial alterations associated with GDM.

Crusell et al. [2] gathered stool samples from children born to mothers with (n = 43) and without GDM (n = 82) during the first week of life and again at a mean age of 9 months. The gut microbiome was characterized through 16S rRNA (V1–V2) gene amplicon sequencing. Differences in diversity and synthesis were assessed according to the GDM state of the mother, while taking into consideration possible confounding factors, such as mode of delivery, perinatal antibiotic therapy, nutrition, and the gender of the infant. Children born to mothers with GDM presented with a differentiated synthesis of the gut microbiome, not only during the first week of life but also at 9 months, at higher taxonomic levels and operational taxonomic unit (OTU) levels. They showed that adjusting for mode of delivery, perinatal antibiotic exposure, and the infant’s gender affected the study’s results. However, even after adjusting for these confounding factors, they continued to notice statistically important associations between the mother’s GDM status and the children’s gut microbiome synthesis. This finding underlines the impact of the mother’s metabolic health during pregnancy in the formation of their offspring’s gut microbiome. Hu et al. [33], aimed to evaluate the meconium’s microbiome diversity and determine whether the bacteria community is defined by the mother’s diabetes status. They selected the first intestinal secretion (meconium) from 23 neonates, categorized according to the mother’s diabetes status; four mothers had pre-existing type 2 diabetes (PDM), including one mother with dizygotic newborns, five had GDM, and thirteen did not have diabetes. Among the meconium samples, classification analyses suggested that the overall bacterial content significantly differed according to the maternal diabetes situation, with the microbiome in the PDM group presenting higher α-diversity compared to the groups without diabetes or the GDM group. The type of delivery did not seem to be associated with changes in the gut microbiome. A regression analysis showed that the strongest predictor for the synthesis of the meconium microbiota was PDM. Specifically, the mothers’ PDM was associated with relative abundance of the *Bacteroidetes* (phyla) and *Parabacteroides* (genus).

Huang, L. et al. [34] compared the gut microbiota among women with GDM, distinguishing between those with successful and unsuccessful disease control through diet (groups GDM-S and GDM-U, respectively) at the time of diagnosis and before delivery. They also compared the gut microflora in newborns of mothers with GDM, with and without successful dietary control. The relevance between the maternal glycemic profile and the gut microflora was also defined. Neonatal Clostridiales was lower in the first feces of the GDM-U group. Additionally, a significantly higher F/B ratio was observed not only in the meconium but also in the first stool of the GDM-U group. Interestingly, the synthesis of gut microflora in both mothers and neonates was associated with the maternal glycemic profile. This study shows that early dysbiosis of the gut is an indicator of diet control failure in mothers with GDM. The gut dysbiosis is associated with dysbiosis in their neonates, which may increase the risk of type 2 diabetes in their descendants later in life. Therefore, analyzing the gut microbiota in women with GDM at the time of diagnosis is beneficial for assessing the risk of unsuccessful dietary modification.

One year later, the same group published a study [35] aimed at identifying the role of maternal gut microflora as an indicator for the need for insulin in patients with GDM and determining the effect of insulin therapy on the composition of gut microflora in mothers with GDM and their neonates. A total of 71 pregnant women were enrolled in the study, including 38 participants with GDM and 33 without GDM. During the monitoring period, 8 out of the 38 pregnant women with GDM required insulin therapy (GDM-I group), while 30 out of the 38 GDM cases achieved glycemic control solely with diet (GDM-D group). Blood and fecal samples from the mothers were collected at the time of GDM diagnosis (pre-therapy, 24 to 28 weeks of pregnancy) and before delivery (post-therapy, 37 weeks of pregnancy). Meconium and first feces samples from the newborns were also collected. Changes in neonatal gut microbiota were observed in the meconium and first feces. The F/B ratio in neonates was higher in both meconium and first feces of the GDM-D group compared to the other two groups. Interestingly, a significant reduction in *Enterobacteriaceae* was found in the first feces of the GDM-I group compared to the meconium of the same group. However, *Eubacteria*, *Clostridiales*, *Lactobacillales*, *Bacteroidetes*, and *Enterobacteriaceae* showed no differences between the groups in both meconium and first feces. These results indicate that there was an increased F/B ratio in neonates of mothers with GDM who did not receive insulin therapy, consistent with the F/B ratio of mothers before delivery. Therefore, it is likely that the composition of maternal gut microbiota could have been transferred to the neonates. An association was observed between maternal glycemic status and neonatal gut microflora composition.

In 2022, results from a study by Song et al. [41] were published, aiming to investigate the relationship between gut flora and GDM and increased body weight (overweight). Thirty-two pregnant women aged 25–35 years were enrolled in the evaluation, including 15 normal-weight pregnant women (NG_NO group), 6 pregnant women with GDM only (G_NO group), and 7 pregnant women with only increased body weight (NG_O group). Fecal samples were collected from the pregnant women at 24 and 37 weeks of pregnancy, as well as meconium from the newborns. The variable region v3–v4 of the 16S rRNA of the gut flora was sequenced and analyzed bioinformatically using the Illumina MiSeq PE300 sequencing platform. The relative distribution of neonatal gut flora at the phylum level was significantly different from that of their mothers. The characteristic gut microbes of the neonates in the G_NO group were *g_Diaphorobacter*, while in neonates of the NG_O group, they were *Nocardiaceae* (*f_Nocardioidaceae*). Additionally, the results showed significant differences in gut flora among the group of normal-weight pregnant women, mothers with normal body weight, simply overweight pregnant women, and overweight pregnant women with GDM. There were significant differences in the composition structure (β- diversity) of the gut flora between pregnant women and their newborns in each group. Correlation analysis showed that the birth weight of the newborns was positively associated with *Actinomycetes* (*Actinomyces*), *Bacteroides* (*Faecalibacterium*), and *microbacillus* (*Dialister*) and negatively associated with *Rolston* (*Ralstonia*).

Ponzo et al. [37] found that infants of mothers with GDM exhibited a higher relative abundance of pro-inflammatory bacterial classes and lower β-diversity compared to infants of healthy mothers. Wang et al. [44] found an increase in the number of lactic acid bacteria in the meconium of newborns from mothers with GDM, indicating that certain specific bacteria colonizing an infant’s gut may be influenced by the mother’s GDM. Su et al. [42] found differences in the gut microbiome between newborns born to mothers with GDM and the control group. The gut microbiome of infants of mothers with GDM showed lower β- diversity compared to the control group. At the phylum level, the abundance of *Proteobacteria* and *Actinobacteria* increased while *Bacteroidetes* decreased in the GDM group. Additionally, some unique gut microbes belonging to the phyla *Proteobacteria*, *Firmicutes*, and *Actinobacteria* were found in the fecal samples of healthy infants but were absent in those with GDM. At the genus level, the number of *Prevotella* and *Lactobacillus* decreased in newborns from mothers with GDM. Correlation analysis showed that maternal fasting blood glucose levels were positively correlated with the relative abundance of the phylum *Actinobacteria* and the genus *Acinetobacter*, but negatively correlated with the relative abundance of *Bacteroidetes* and the genus *Prevotella*.

Soderborg et al. [39] examined whether GDM alone or in combination with maternal obesity during pregnancy leads to early microbial colonization of the offspring’s gut in a carefully selected cohort of full-term newborns, who were born vaginally, mostly breastfed, and had no history of prenatal or postnatal antibiotic exposure. They observed differences in the abundance of 26 microbial categories in the feces of newborns born to mothers with GDM, 14 of which showed persistent differential abundance after adjusting for pre-pregnancy body mass index. Given that obesity is the main driver of insulin resistance, the results of this study suggest that fetal exposure to GDM, in addition to insulin resistance, contributes to altered microbial colonization during neonatal and infant ages. Key pioneering classes of the gut microbiome, including potentially important classes for establishing neonatal immunity such as *Lactobacillus*, *Flavonifractor*, *Erysipelotrichaceae*, and unspecified families in *Gammaproteobacteria*, were significantly reduced in newborns of mothers with GDM. GDM was associated with an increase in microbes involved in the suppression of early immune cell function (*Phascolarctobacterium*). There were no differences in the levels of short-chain fatty acids (SCFAs) in the infants’ stool according to maternal phenotype. However, significant correlations were found between microbial abundance and SCFA levels in the neonates. Their results suggest that GDM, both alone and in association with maternal overweight/obesity, uniquely affects the seeding of specific patterns of the infant microbiota, setting the background for future risks of inflammatory and metabolic diseases.

Contrary to all previous studies, Sililas, P. et al. [38] did not observe differences in the meconium and early stool microbiome in newborns of mothers with or without GDM. The main finding of this study is that gut microbiota quantities during pregnancy with GDM did not significantly differ from those in pregnancies without GDM, which contrasts with previous studies suggesting that gut microbiota dysbiosis may play a significant role in the development of GDM. According to the authors, this finding could be attributed to interventions aimed at controlling blood sugar, either through diet or insulin in cases of GDM. This intervention may eliminate the natural course of gut microbiota-related effects.

Guzzardi et al. [45] reported similar conclusions. They examined the associations between gut microbiota and cognitive development during infancy, and their connection to maternal obesity. In groups of children from the Pisa Birth Cohort (PISAC), the authors analyzed gut microbiota composition, using 16S rRNA gene sequencing, in samples of the first meconium and stools collected at 3, 6, 12, 24, and 36 months of age. The relationship of the microbiota with maternal obesity or diabetes during pregnancy and cognitive development of the offspring, as measured from 6 to 60 months using the Griffiths Mental Developmental Scales (GMDS), was investigated. The gut microbiota profile of the offspring was not associated with maternal GDM at any age. Only a few bacteria showed differences in abundance between the groups, but these differences were no longer statistically significant after multiple testing correction.

### 3.3. The Correlation of GDM with Dynamic Changes in the Offspring’s Gut Microbiota over Time

Several studies indicate that early colonization is essential for the development and maturation of gut microflora. In this perspective, research teams attempted to assess the maturation of gut microflora in infants born to mothers with GDM over time, compared to the control group (Table 3).

In 2023, a research team led by Song et al. [40] presented results from a cohort study aimed at prospectively evaluating the impact of GDM on the gut microbiota of infants at ages 1 and 6 months old (phase M1 and phase M6, respectively), as well as the dynamic changes in the infants’ gut microflora between 1 and 6 months of age. Although no significant differences in diversity and composition were observed between the GDM and non-GDM groups in phase M1, differential structures and compositions were noted in phase M6 between the two groups. Infants born to mothers with GDM showed lower diversity levels in their gut microbiota. Moreover, the dynamic changes in α-diversity from phase M1 to M6 were also significantly different depending on the GDM status. Additionally, altered gut bacteria in the GDM group were correlated with infant growth.

Valdez-Palomares et al. [43] analyzed fecal samples from infants aged 0–6 months, 7–12 months, and 13–30 months who were exposed or not exposed to GDM during pregnancy. Their aim was to identify taxonomic changes associated with age and GDM and assess the maturity of the infants’ gut microflora born to mothers with GDM compared to those without GDM. In that study, no changes in the composition of the gut microbiota were observed when comparing children exposed to GDM in comparison to those not exposed to GDM. However, when groups were analyzed by age, the offspring of mothers with GDM maintained lower α-diversity. As mentioned earlier, the gut microbiota in early childhood (2 years old) undergoes changes over time [46], both at taxonomic levels and in measures of α and β diversity [47]. Offspring not exposed to GDM showed a continuous growth and maturation of the intestinal microbiota in early life, unlike offspring from mothers exposed to GDM who exhibited lower α-diversity. This lower α-diversity has been associated with delayed microbial maturation and subsequent adverse health outcomes, including atopy and allergic diseases [48,49]. This finding is consistent with other studies indicating that GDM is linked to lower diversity [2,40].

Valdez-Palomares et al. [43] found that α-diversity remained reduced in offspring of mothers with GDM and did not show the recovery observed at nine months by Crusell et al. [2]. Furthermore, the gut microbiota of overweight children has lower diversity and richness [50], suggesting that in children with GDM, the immaturity of the gut microbiota could be a decisive factor in future obesity development [51]. Several studies indicate that early colonization is crucial for the creation and maturation of the gut microbiota.

The development of early childhood allergies is an increasing public health concern associated significantly with GDM [52]. Modified human milk glucobiome can profoundly influence the infant gut microbiota and the development of immune tolerance, mediated by intestinal Treg cells in infants born to mothers with GDM.

Li, X. et al. [36] found that compared to healthy Chinese mothers, mothers with GDM had significantly lower levels of total and specific HMOs in their colostrum, which subsequently increased with prolonged lactation. This change in HMO profiles significantly delayed the colonization by *Lactobacillus* and *Bifidobacterium* species in their breastfeeding infants, resulting in a distinct microbial structure and gut metabolism. These findings clearly indicate a delay in the colonization of the intestinal system of breastfeeding infants born to mothers with GDM by certain bacteria. Infants, breastfed by mothers without GDM, showed early dominance and colonization by *Lactobacillus* and *Bifidobacterium species* by day 6 and day 42 of lactation, respectively. By contrast, in the gut system of breastfeeding infants born to mothers with GDM, the Lactobacillus and Bifidobacterium species became dominant after days 42 and 90, respectively. These results suggest that besides the vertical transmission of modified gut microbiota from mothers with GDM, the low concentration of oligosaccharides in maternal milk during the initial lactation period in mothers with GDM may significantly suppress the proliferative capacity of *Lactobacillus* and *Bifidobacterium species* in the infant gut. Furthermore, the results of this study indicate that as the concentration of oligosaccharides in the breast milk of mothers with GDM increased by day 42, there was also a significant increase in their proliferative capacity as well as their ability to utilize oligosaccharides. Additionally, the quantities of other bacterial groups in the infants’ gut were also significantly correlated with the oligosaccharide content in maternal milk, indicating a strong influence of oligosaccharides on the formation and functions of the neonatal microbiota.

### 3.4. The Correlation of Gut Microbiota with the Outcomes of Offspring from Mothers with GDM

The correlation of gut microbiota with the outcomes of offspring from mothers with GDM is a research field that underscores the importance of microbial communities in infant development and health. Few studies were identified in our systematic review that specifically aimed to correlate gut microbiota with the outcomes of offspring from mothers with GDM. In the study by Song et al. [40], a correlation analysis was conducted aiming at the further exploration of potential correlations between infant anthropometric parameters and altered gut microbiota in the GDM group. Seventy-three mother–infant pairs were evaluated, including 34 with GDM and 39 without GDM. Overall, the family *Coriobacteriaceae* and its maternal class *Coriobacteriia* were negatively correlated with Z-score weight for age (ZWFA), Z-score length for age (ZWFL), Z-score body mass index for age (ZBMI), and Z-score head circumference for age (ZHeadC) in infants at 6 months of age. Additionally, the genus *Ralstonia* and the family *Oxalobacteraceae* were also negatively associated with head circumference. These findings suggest that disruptions in gut microbiota are significantly linked to infant growth.

To explore whether the potential correlation between GDM and infant body mass index (BMI) in early childhood is influenced by meconium microbiota, Zhu et al. [6] recruited 120 mothers (60 healthy women and 60 with GDM) and their neonates. Meconium was collected from neonates within hours after birth, and the sequence was analyzed using 16S rRNA sequencing. The BMI scores of the children were measured at 12 months of age. The results revealed that infants born to mothers with GDM had increased Z-score BMI at 12 months and that the β -diversity of their meconium microbiota was reduced. Several genera were observed to be significantly different between GDM and control groups. The abundance of the genus *Burkholderia-Caballeronia-Paraburkholderia* and an unclassified genus of the family *Enterobacteriaceae* in neonates born to healthy mothers was negatively associated with infant BMI using regression analysis. The reduced abundance in the GDM group was negatively correlated with BMI at 12 months. This study provided evidence for correlations between maternal GDM, meconium microbiota, and infant BMI. GDM was shown to affect infant BMI, mediated through gut microbiota. Interventions targeting gut microbiota may represent a novel approach towards reducing the risk of childhood obesity caused by GDM.

Li, X. et al. [36], created a food allergy model to further explore the impact of changes in gut microbiota composition, the organ of immune tolerance in mice. Their results showed that levels of pro-inflammatory factors such as IL-4, IFN-γ and TGF-β were significantly higher in the GDM + ovalbumin group (mice immunized with subcutaneous injection of 100 μg of ovalbumin, +OVA) compared to the control + OVA group. Additionally, the ratio of Treg cells, specifically RORγt+ Treg cells, in Peyer’s patches was significantly reduced in the GDM + OVA group. In conclusion, this study revealed changes in human milk oligosaccharide profiles in mothers with GDM and assessed the potential impact of such changes on the development of neonatal gut microbiota and immune tolerance mediated by RORγt+ Treg cells. These findings may provide motivation for studies in human populations.

## 4. Discussion

In the current systematic review, significant changes in the intestinal microbiota of offspring from mothers with GDM were highlighted. These changes mostly include reduced α and β- diversity and alterations in the relative abundance of certain bacterial species. Specifically, the microbiota of neonates born to mothers with GDM significantly differs from that of neonates born to healthy mothers, showing an increased abundance of pro-inflammatory bacteria and reduced diversity.

Indicatively, Chen et al. [1] found that the microbial communities in the meconium were significantly altered in neonates born to mothers with GDM, with important changes in α and β- diversity. Crusell et al. [2] observed differences in the composition of children’s microbiota from mothers with GDM both in the first week of life and at 9 months, considering various confounding factors. Similar findings are reported by Hu et al. [33], showing that maternal diabetes affects the composition of meconium microbiota. Additionally, Huang et al. [35] reported that early gut dysbiosis in neonates is associated with poor dietary control in mothers with GDM, increasing the risk of type 2 diabetes in their offspring. Studies by Song et al. [40,41] and Ponzo et al. [37] confirm changes in the relative abundance and differentiated composition of gut flora.

However, in only two studies, no significant differences were found in the gut microbiota between neonates born to mothers with or without GDM, with the authors suggesting that these findings may be due to interventions for glucose control in GDM cases, which could potentially mitigate natural impacts on the microbiota [38,45]. Another study reported that a statistically significant higher F/B ratio was observed both in meconium and in the first feces of neonates from mothers with GDM who did not receive insulin therapy, whereas no such difference was found in neonates from mothers with GDM who received insulin therapy and mothers without GDM. At the same time, a decrease in *Enterobacteriaceae* was noted in the feces of neonates from mothers with GDM who received insulin therapy. *Enterobacteriaceae* is a Gram-negative bacterium that produces lipopolysaccharides and has been associated with low-grade systemic inflammation [53]. These findings suggest that the composition of maternal gut microbiota may be transmitted to neonates and further indicate a potentially transferable benefit of maternal insulin therapy to neonates, possibly mediated through a reduction in inflammation due to insulin [35].

Lately, the importance of the maternal microbiota has been showcased due to the revelation of important changes that happen to its composition during pregnancy [54]. The changes that happen in the genital tract, the gut, the mouth, etc., and their link to gestational complications have not been fully studied [55,56]. A profound change has been noted to the gut microbiota from the first to the third trimester, with the microbiota undergoing a dramatic reconstruction when it comes to variety and abundance [57]. During gestation, the composition of the enteric microbiota during the first trimester (T1) is similar to healthy pregnant women without GDM and differentiates gradually during the second trimester (T2). The dominant species is *Firmicutes,* mainly *Clostridiales*, whereas there are fewer *Bacteroidetes* [58].

During the midst of gestation, T2, the abundance of *Bifidobacteriaceae* and *Enterobacteriaceae* increase. However, during the third trimester (T3), the most important changes to the microbiota of the mother’s gut is noticed, and it is similar to the gut microbiota of those with metabolic syndrome or obesity [54]. The level of bacteria that produce butyric acid, like *Faecalibacterium*, which has anti-inflammatory properties, are reduced during this period [59]. Furthermore, the ratio between *Firmicutes* and *Bacteroidetes* fluctuates, with a higher number of *Firmicutes* and a smaller ratio of *Bacteroidetes* noticed in the gut microbiota. A greater number of *Streptococcus* and *Enterobacteriaceae* has been noted during T3, which are also the primary conquerors of the neonatal gut, and this shows the possible transition of the maternal gut microbiota to the neonate’s gut microbiota [60]. Hormones such as estrogen and progesterone play a critical role in the preservation and control of gestation [61]. The first hormone that changes during pregnancy is estrogen, which activates the change in the gut microbiota. The gut microbiota contains a specific number of bacteria that can process estrogen, known as “estrobolome”. Progesterone can change the gut microbiota during pregnancy and can lead to an increased number of *Bifidobacterium species* [62,63,64]. However, little is known about the relationship between hormones and gut microbiota during gestation.

According to the widespread accepted concept of a sterile in utero environment, the neonatal microbiota is established during and after labor. However, many new studies that use the latest sequencing technologies have shown that neither the fetus nor the placenta or amniotic fluid are sterile, and the microbial colonization of the infant’s gut starts while in the uterus. The existence of a microflora in the placenta is still debated [65,66]. The human placenta has historically been considered sterile, and microbial colonization has been linked to an adverse gestational outcome. On the other hand, recent studies using DNA sequencing have mentioned the existence of a microflora in human placentas [67]. However, this microflora could represent DNA fragments or contamination that is linked to the act of childbirth itself [68,69].

Only after showing a legit and viable trace of bacterial DNA from caesarian sections through a sterile protocol with technical checks and positive data from cultures can we evaluate the level in which the maternal immune system can tolerate these bacteria without causing an adverse immune reaction and whether or not the existence of this bacteria looks like coexistence or contamination. Finally, placental microflora can exist or not exist, but it is clear that the efforts to preserve sterility and avoid contamination have not been successful, as the vast majority of DNA sequencing from placental samples can be affected by various types of contamination. Therefore, methodologies of DNA definition require major improvement so as to be able to confirm the existence of a placental microflora, because, as of the present, the gene sequence 16s rRNA seems to lack the ability to distinguish between a low biomass microflora and DNA contamination [65].

Additionally, the detection of specific meconium OTUs across multiple maternal samples, along with the resemblance between the meconium microbiota and that found in the amniotic fluid, suggests that the meconium microbiota may have originated from diverse maternal origins. Notably, the amniotic sac seems to have exerted a more substantial influence than other maternal body sites in contributing to the meconium microbiota [70]. Since the fetus ingests amniotic fluid during the second and third trimesters, it is anticipated that the microbiomes of the amniotic fluid and meconium would significantly overlap. Research has shown that the meconium microbiome is more closely related to the microbiome of amniotic fluid than to the microbiomes of maternal feces, placenta, colostrum, or infant feces [71]. Based on this information, it has been suggested that gradual colonization of the gut microbiome may begin prenatally with the unique microbiota found in the placenta and amniotic fluid. This maternal–offspring microbial connection is further sustained postnatally through the microbes present in breast milk [72].

The first source for the neonate’s gut microbiota is the mother with the highest exposure happening during labor and the perinatal period through vertical and horizontal transfer [73,74]. Microorganisms can grow during gestation, and there can be exposure in the uterus, which can be followed by important microbial colonization during labor [75,76].

The changes in microbiota are related to the delivery mode, perinatal antibiotic exposure, diet, and other confounding factors. Early colonization of intestinal bacteria in infants typically occurs at birth. During the first few days, only a few groups of allogeneic microbes, unrelated to dietary sources, settle in the intestines and become more stable in the first week of life. At that time, potentially anaerobic microbes belonging to *Enterobacteriaceae*, *Streptococcus*, *Staphylococcus*, and *Enterococcus* families are already present, mainly due to initial oxygen exposure in the neonatal gut [77]. *Escherichia coli*, *Enterococcus faecium*, and *Enterococcus faecalis* are the most represented species among the early colonizers. With the gradual increase in oxygen consumption by potentially anaerobic microbes, an anaerobic environment is created in the intestine, leading to an increase in obligate anaerobes such as *Bifidobacterium*, *Bacteroides*, and *Clostridium*.

Upon the introduction of solid foods, the colonization and diversity of bacteria in the intestine undergo continuous changes, with one of the most notable features being the increase in *Bacteroide* numbers. Among the intestinal bacteria in the early stages of healthy infants, *Bifidobacterium* predominates. *Bifidobacterium* appears on the 3rd–4th day after birth, gradually increases, and peaks in the first year. Over time, the quantity of *Bifidobacterium* begins to decrease in the second year [66], while other species of intestinal microbiota start to diversify, making the infant gut microbial community more varied. After the age of 3 years, the gut microbiota environment changes rapidly, stabilizing in composition and beginning to resemble that of adults, dominated by *Firmicutes* and *Bacteroidetes* [78].

The “normal” gut microbiota of infants develops through colonization by facultative anaerobes and later by obligate anaerobes such as *Bifidobacterium*, *Bacteroides*, and *Clostridium* [79]. These anaerobic microorganisms produce polysaccharides that mediate microbiota colonization, regulate the immune system, and interact with the host–gut crosstalk. For example, high levels of *Clostridium* in the infant’s gut are considered pathogenic and unhealthy [80,81]. Both *Bifidobacteria* and *Lactobacilli* contribute to both innate and acquired immune responses in healthy neonates [37,82]. According to research, there is a correlation between low levels of *Bifidobacteria* in early stool samples and a higher risk of non-communicable diseases later in life, such as atopic diseases and obesity. The presence of *Bifidobacteria* in the adult intestinal microbiota is minimal, indicating that *Bifidobacteria* are specifically detectable in early life. The F/B ratio, often used as an indicator of dysbiosis [83], was found to be elevated in the offspring of mothers with GDM, suggesting a potential disturbance in microbiota balance [35].

The gut microbiota is linked to the regulation of metabolic and inflammatory axes in the liver, muscles, and brain through host pathways. Intestinal microorganisms produce various metabolites such as short-chain fatty acids that act through host receptors and signaling pathways to influence metabolic and immune processes. This bidirectional communication is crucial for maintaining homeostasis and regulating inflammation in the body, emphasizing the importance of a balanced relationship between the intestinal microbiota and various host organs [77,84,85]. Dysbiosis, or the imbalance of infant gut microbiota, can be facilitated by early exposure to environmental factors such as bacteria and viruses, which can also alter the host’s microbiota. This dysbiosis of the microbiota has long-term implications for the host’s metabolism, leading to metabolic changes, especially in type 1 diabetes, autoimmune diseases, and obesity. Human and animal studies examining the possibility of causal links in disease programming indicate that dysbiosis of the gut microbiota negatively affects metabolic health, triggering the onset of cardiometabolic diseases later in life [27]. According to the “developmental origins of health and disease” hypothesis, increasing evidence supports the theory that exposure to prenatal metabolic disorders during embryonic development may contribute to health outcomes in offspring [86]. It is known that the maternal environment influences offspring health. The gut microbiota of the neonate is dramatically influenced by maternal health and pregnancy conditions, participating in the developmental programming of neonates [77,87,88].

Research has shown that maternal microbiota of the oral cavity, vagina, and gut can be modified during pregnancies complicated by gestational GDM [44]. Changes in gut microbiota have been observed in pregnancies complicated by GDM during the first, second, and third trimesters, extending up to 8 months postpartum [81,89,90,91]. Several studies have indicated correlations between the relative abundance of certain gut bacteria and carbohydrate metabolism. Additionally, associations have been found between the dysbiosis of vaginal microbiota during pregnancy and complications such as preterm birth and low birth weight [3,92,93,94,95]. The abnormal composition of neonatal oral microbiota has also been linked to maternal GDM [96]. Studies have highlighted the enrichment of specific bacterial species in the oral microbiota of neonates born to mothers with GDM. Furthermore, there is evidence that differences in the composition of maternal milk contribute to changes in gut microbiota composition in breastfed infants of obese mothers [44].

Maternal microbiota, whether intestinal, vaginal, or breast milk-associated, is closely linked to infant health [97,98]. Dysbiosis or changes in maternal microbiota can lead to adverse pregnancy outcomes [99,100]. Various factors influence the vertical transmission of maternal microbiota to the newborn, such as maternal age at birth, delivery mode, feeding method, maternal diet, and other environmental factors. Maternal milk composition plays a significant role in shaping infants’ gut microbiota [101]. The delivery mode can influence the initial colonization of gut microbes and also affect the development of microbiota composition in infants, similar to the effects of perinatal exposure to antibiotics [78,102,103,104,105,106].

However, from our systematic review, several researchers have observed statistically significant correlations between GDM status and gut microbiota composition in children, even after adjusting for these confounding factors [2,35,36,37]. This finding underlines the importance of maternal metabolic health during pregnancy in shaping offspring gut microbiota. Furthermore, GDM has been associated not only with the structure and composition of the gut microbiota community at a specific time point but also with differential changes from birth to infancy [2]. Altered colonization of infant gut microbiota born to mothers with GDM may impact their development. These findings highlight the critical impact of GDM on early life gut microbiota formation and on infant growth and development [2,6,36].

The contribution of maternal gut microbiota and breast milk microbiota, separately or both together, to the colonization of the infant’s gut microbiota, and whether other factors in the infant’s gut environment predispose them to disorders/dysbiosis in cases of offspring of mothers with GDM, remain unclear. Further research using large prospective cohort studies of the mother–neonate duo is essential to understand how these early microbiota changes may interact with host factors to promote different aspects of immune response and chronic diseases in offspring. Early patterns of microbial succession during the first year of life have been associated with susceptibility to immune-related diseases later in life [51]. This represents an important challenge but also a unique opportunity to discover pathways involved in the developmental programming of the neonate. Understanding the mechanisms through which GDM influences offspring microbiota offers opportunities for developing prevention strategies. Interventions such as improving diet during pregnancy and using probiotics may contribute to maintaining a healthy microbiota in offspring.

Despite significant changes observed in the infant gut microbiota due to GDM in this systematic review, various limitations hinder clear conclusions from being drawn. Heterogeneity among studies, including differences in participant ethnicity, diverse criteria for GDM diagnosis, variations in participant characteristics, and the timing of microbiota analysis, have prevented meta-analyses from identifying or excluding statistically significant differences.

Furthermore, when evaluating our review’s study results, it is crucial to recognize that some studies use meconium samples while others use fecal samples for microbiota analysis. These sample types are not comparable due to their distinct origin conditions and composition. Microorganisms from both maternal and environmental sources rapidly colonize the newborn’s gut at birth. As previously mentioned, the development and maturation of gut microbiota are dynamic processes involving interactions among significant bacterial groups starting within hours after delivery [107,108]. Another key factor influencing early colonization is the type of feed—breastfed or formula-fed. Meconium, the newborn’s first stool, should be compared only with other meconium samples to accurately understand the factors that influence fetal microbiota composition during pregnancy and birth. Conversely, fecal samples collected post breastfeeding initiation or introduction of solid foods exhibit different bacterial compositions, reflecting varied stages and mechanisms of microbiota transmission from mother to child. These samples depict a different developmental trajectory and microbiota changes as the child’s diet and others environmental factors evolve. Hence, a precise comparison of samples is essential for comprehending microbiota transmission and development in children. Distinguishing between meconium and fecal samples is critical for accurately assessing the development of fetal–neonatal microbiota and anticipating changes in bacterial composition due to dietary and other influences.

Although it is often assumed that fecal samples can serve as a proxy for the entire gut microbiome, recent research has questioned this assumption [109,110,111]. Stool consistency, chemical composition, and physical environment within the gastrointestinal tract vary significantly. Studies have shown that an individual’s gut microbiome composition can differ over time and with different sampling methods, indicating that relying on a single fecal sample may not provide an accurate representation of the whole gut microbiome. Additionally, gut microbiome profiles obtained from blood plasma signatures differ from those derived from fecal samples, further suggesting that fecal samples may not fully capture the complexity of the gut microbiome [112,113].

It is important to note that the composition of fecal microbiota differs from that of microbiota found in other parts of the digestive tract [114]. To understand these differences, several studies have evaluated the biogeography of the gut microbiota. Although differences exist, it has been reported that the microbial contents of the large intestine (colon), which has a reduced transit time and high nutrient availability, correlate with feces in terms of species diversity and bacterial abundance (10^11^ to 10^12^ bacteria per gram). By contrast, the microbial content of the small intestine (ileum, jejunum, and duodenum) contains fewer microbial nutrients, is exposed to bile acids and pancreatic enzymes, and has a shorter transit time, resulting in decreased diversity and abundance (10^4^ to 10^8^ bacteria per gram) [115,116,117]. The microbial content in the stomach is markedly different, with only low diversity and abundance (<10^4^ bacteria per gram) due to the extreme acidic conditions [118]. Additionally, the microbiota associated with the outer mucus layer of the colonic mucosa differs from the luminal microbiota in the same compartment, whether in healthy or diseased states [116]. The inner mucus layer and crypts of the mucosa, which contain intestinal stem cells, were once thought to be devoid of bacteria; however, recent discoveries have identified crypt-associated microbiota in mice [119]. Despite these limitations, most gut microbiota studies use stool samples, which are easy to collect noninvasively and are considered reflective of overall variations in colonic microbiota. The exception is when surgical procedures are part of the study, which allows access to luminal or mucosal compartment-specific microbiota [114].

## 5. Conclusions

The mechanisms through which the microbiota of neonates and infants change in response to GDM are not well understood and need to be evaluated in future research. Future directions in studying maternal and infant microbiota will open new avenues. Recent advances in metagenomics, metatranscriptomics analysis, and bioinformatics will enable larger prospective studies, which will help understand the evolution of gut microbiota across different stages of pregnancy, delivery, and the perinatal period, offering new preventive and therapeutic approaches. Further study of GDM’s role in the initial colonization of microbiota, how maternal microbiota may influence fetal metabolic programming, and how infant microbiota may lead to the future development of obesity and glucose intolerance is crucial. Future studies should include larger sample sizes, an appropriate collection of potential confounding factors, an evaluation of maternal interventions for GDM, and longitudinal designs to better understand possible connections with long-term detrimental consequences, such as obesity and impaired glucose tolerance. Understanding the mechanisms leading to microbiota disturbance in the offspring of mothers with GDM can lead to the development of new strategies for the prevention and management of the impacts of this condition on infant health in the long term.

## Figures and Tables

**Table 1:** Characteristics of studies included in the systematic review.

Author, Year, Location	Study Design	GDM Definition	Population (n)	I-GDM	I-Non GDM	GA/BW of I-GDM (Weeks, g)	GA/BW of I-Non GDM (Weeks, g)	Cesarean Delivery I-GDM (n, %)	Vaginal Delivery I-GDM (n, %)	Cesarean Delivery I-Non GDM (n, %)	Vaginal Delivery I-Non GDM (n,%)	Timing of Microbiome Analysis	Sample Type	Method of Microbial Analysis	Study Results
Chen et al. [1], 2021, China	Cross-sectional	The WHO criteria	418	147	271	3329.22 ± 347.42	3511.63 ± 425.40	70 (25.83)	201 (74.17)	60 (40.82)	87 (59.18)	Within the first few hours of life	Meconium	16S rRNA gene sequencing (V3 region).	Significant reduction in α-diversity in I-GDM compared to I-non GDM was observed.
Crusell et al. [2], 2020, Denmark	Cohort	International Association of Diabetes Pregnancy Study Groups, 2010	125	43	82	39.4 ± 1.5/3559 ± 535.6	39.7 ± 1.7/3690 ± 402.0	9 (20.9)	34 (79.1)	11 (13.4)	71 (86.6)	The first week of life and at an average age of 9 months	Fecal samples	16S rRNA gene amplicon sequencing (V1–V2 region).	Lower richness of the gut microbiota in GDM neonates compared to those born to mothers without GDM were founded.
Guzzardi et al. [45], 2022, Italy	Cohort	The criteria of the American Diabetes Association (ADA)	90 neonates–other pairs; 79 meconium samples	NR	NR	NR	NR	NR	NR	NR	First-pass meconium samples, and at the age of 3, 6, 12, and 36 months	Meconium and stool samples	16S rRNA genes sequencing (V3–V4 region)	NR	The gut microbiota profiles of the offspring did not show any correlation with maternal GDM at any age.
Hu et al. [33], 2013, USA	Case–control	NR	23	10 (4 mothers with pre-gestational type 2 diabetes mellitus	13	2600–3800	1645–4060	6 (60)	4 (40)	4 (30.8)	9 (69.2)	2 h and 48 h after birth	Meconium	16S rRNA sequencing (V3–V4 region)	In the meconium samples, taxonomic analyses indicated that the overall bacterial content significantly differed based on maternal diabetes status.
Huang et al., 2021 [38], Thailand	Observational	National Diabetes Data Group criteria	79	I-GDM-S (n = 28) and I-GDM-U (n = 13)	38	NR/3120 ± 73.67 for I-GDM-S and 3141 ± 73.52 for I-GDM-U	NR/3087 ± 52.81	12 (43) I-GDM-S and 4 (31) I-GDM-U	16 (57) I-GDM-S and 9 (69) I-GDM-U	9 (24)	29 (76)	NR	Meconium and the first feces	16S rRNA gene sequencing (NR region).	*Firmicutes*-to-*Bacteroidetes* F:B ratio was observed significantly higher in both meconium and the first feces of the I-GDM vs. I-non GDM group, and I-GDM-U vs. I-GDM-S group
Huang et al. [35], 2022, Thailand	Observational	National Diabetes Data Group criteria	71	38	33	NR	NR	15 (39.4)	23 (60.5)	8 (24.2)	25 (75.8)	Within 24 and 48 h after birth	Meconium and first feces	16S rRNA gene sequencing (NR region).	Insulin therapy altered the composition of maternal gut microbiota, potentially influencing the microbiota transferred to the mothers’ newborns.
Li et al. [36], 2023, China	Observational	If any of the three blood glucose levels measured during pregnancy (fasting/1 h postprandial/2 h postprandial blood glucose) was >10.0 mmol/L	130	74	56	39.97 ± 2.84/3530 ± 363	40.41 ± 1.93/3360 ± 382	43 (59.7)	29 (40.3)	34 (61.8)	21 (38.2)	At 5, 42, and 90 days of life	Fecal samples	16S rRNA sequencing, taxonomic assignment and diversity analysis (V3–V4 region).	A significant delay in the colonization of *Lactobacillus* and *Bifidobacterium* spp. in breast-fed infants born to mothers with GDM was observed, resulting in a distinct gut microbial structure and metabolome.
Ponzo, V. et al. [37], 2019, Italy	Cohort	International guidelines	48	29	19	NR	NR	NR	NR	NR	NR	3–5 day of life	Fecal samples (following meconium expulsion)	16S rRNA sequencing (V3–V4 region).	Infants born to mothers with GDM exhibited a higher relative abundance of proinflammatory taxa compared to infants born to healthy women.
Sililas et al. [38]. 2021, Thailand	Longitudinal	National Diabetes Data Group (NDDG) criteria	88	49	39	38.3 ± 1.0/3079 ± 369.9	38.3 ± 1.0/3087 ± 321.3	18 (36.7)	31 (63.3)	9 (23.1)	30 (76.9)	At 24 and 48 h of life	Meconium	Bacterial genomic DNA was isolated from human fecal samples using a commercial DNA extraction kit. The 16S rRNA gene sequencing method was not employed.	There was no difference in neonatal gut microbiota between the groups.
Soderborg et al. [39], 2020, USA	Cohort	Carpenter and Coustan criteria	46	13	33	39.4 ± 0.9/3240 ± 360	40.0 ± 1.0/3350 ± 510	NR	NR	NR	NR	At the age of 2 weeks	Fecal samples	16S rRNA gene amplicon sequencing (V1–V2 region).	GDM, both alone and in conjunction with maternal overweight or obesity, uniquely affects the initial colonization of specific infant microbiota. These patterns may predispose infants to an increased risk of inflammatory and metabolic diseases in the future.
Song et al. [41], 2022, China	Cohort	Chinese Society of Obstetrics and Gynecology Guidelines for the Diagnosis and Management of Gestational Combined Diabetes Mellitus	28 neonates born to 15 normal pregnant women (NG_NO group), 6 pregnant women with GDM alone (G_NO group), and 7 pregnant women with overweight alone (NG_O group)	6	22	NR	NR	NR	NR	NR	NR	First hours of life	Meconium	16s rRNA double-ended sequenced and bioinformatically analyzed (V3–V4 region).	The gut microbiota of pregnant women and their newborns is closely associated with obesity and GDM.
Song et al. [40], 2023, China	Longitudinal	Chinese Society of Obstetrics and Gynecology Guidelines for the Diagnosis and Management of Gestational Combined Diabetes Mellitus	73	34	39	37–42/2500–4000	37–42/2500–4000	NA	NA	NA	NR	1 month of age (“M1 phase”) and at 6 months of age (“M6 phase”)	Fecal samples	16S rRNA gene sequencing (V3–V4 region).	Maternal GDM was associated not only with the community structure and composition of the offspring’s gut microbiota at specific time points but also with differential changes from birth to infancy.
Su et al. [42], 2018, China	Longitudinal	International Association of Diabetes and Pregnancy Study Groups (IADPSG	34	20 (15 neonates born to mothers with GDM treated by diet and exercise (GDM_A1) and 5 neonates born to mother with GDM treated by insulin in combination with diet and exercise (GDM_A2))	14	GDM_A1: 38.53 ± 1.06/3240 ± 550; GDM_A2: 39.00 ± 0.00/3350 ± 310	38.93 ± 0.62/3390 ± 360	NR	NR	NR	NR	Within 24 h of life	Meconium	16S rRNA gene amplicon sequencing (V4 region).	Sequencing and bioinformatics analysis of 16S rRNA revealed that the gut microbiota of newborns with GDM differed from that of control newborns. Taxonomy analyses indicated that the overall bacterial composition varied significantly based on maternal diabetes status, with the microbiome of the GDM group exhibiting lower alpha diversity compared to the control group.
Valdez-Palomares et al. [43], 2024 Mexico	Cross-sectional	International Association of the Diabetes and Pregnancy Study Group criteria	40	14	26	38 (33–40)/2875 ± 923	38 (37–40)/2752 ± 599	10 (71)	4 (29)	18 (69)	6 (31)	0–6 months, 7–12 months, and 13–30 months	Fecal samples	16S rRNA gene (V3–V4 region) QIIME2 and Picrust2	The offspring of mothers with GDM exhibit a distinct taxonomic profile characterized by taxa linked to gut microbiota immaturity.
Wang et al., 2018 [44], China	Cohort	Based on the results of OGTT	140 neonates (83 samples)	NR	NR	NR	NR	NR	NR	NR	NR	Within 24 h of life	Meconium	16S rRNA gene and metagenomic sequencing (V3–V4 region).	Microbiota of neonates whose mothers suffered from GDM differed significantly from that of controls.
Zhu et al. [6], 2022, China	Cohort	International Association of the Diabetes and Pregnancy Study Group criteria	120	60	60	39.26 ± 1.27/3476 ± 400	39.26 ± 1.01/3361 ± 360	44 (73.3)	16 (26.7)	44 (73.3)	16 (26.7)	A few hours after birth	Meconium	16S rRNA genes sequencing (V3 region).	The dysbiosis of the gut microbiome induced by maternal GDM may potentially play a significant role in the increased infant BMI during the first 12 months of life.

Abbreviations: 16S ribosomal RNA, rRNA; birth weight, BW; gestational age, GA; gestational diabetes mellitus, GDM; infants born to mothers with GDM, I-GDM; infants born to mothers with GDM with unsuccessful diet control, I-GDM-U; infants born to mothers with GDM with successful diet control, I-GDM-S; infants from non-GDM mothers, I-non GDM; non reported, NR; not available, NA; oral glucose tolerance test, OGTT.

**Table 2:** The gut microbiota in the offspring of women with gestational diabetes mellitus (GDM).

Author, Year, Location	Population (n)	I-GDM	I-Non GDM	Timing of Microbiome Analysis	Sample Type	Method of Microbial Analysis	Bacteria Depleted in N-GDM	Bacteria Enriched in N-GDM	Study Results
Chen et al. [1], 2021, China	418	147	271	Within the first few hours of life	Meconium	16S rRNA gene sequencing	Phylum: *Proteobacteria*	Phylum *Firmicutes*; Genera: *Rothia Lactobacillus* and *Clostridium sensu*; Family: *Streptococcaceae*	Significant reduction in α-diversity in I-GDM compared to I-non GDM was observed.
Crusell et al. [2], 2020, Denmark	125	43	82	The first week of life and at an average age of 9 months	Fecal samples	16S rRNA gene amplicon sequencing	Phylum *Firmicutes*; Genus: *Veillonella*, *Megasphaera* (*Veilonellaceae*, *Selenomonadales*, and *Negativicutes*), *Subdoligranulum*, *Ethanoligenes*. Phylum *Bacteroidetes*; Genus *Prevotella*, parent family *Prevotellaceae*. Phylum *Actinobacteria*; Genus: *Rothia*, parent family *Micrococcaceae*. During infancy, phylum *Proteobacteria* Genus *Shewanella* and its parent family (*Shewanellaceae*) and order (*Alteromonadales*), as well as genus *Propionibacterium* and its parent family *Propionibacteriaceae* and genus *Collinsella*, within *Actinobacteria* phylum. During infancy, phylum *Firmicutes*, genus *Dorea*	Phylum *Firmicutes*; Genus: *Isobaculum*, the parent family *Carnobacteriaceae*, *Turicibacter*. During infancy, phylum *Proteobacteria* but no subordinate taxa.	Lower richness of the gut microbiota in GDM neonates compared to those born to mothers without GDM was found.
Guzzardi et al. [45], 2022, Italy	90 neonates-mother pairs; 79 meconium samples	NR	NR	Meconium and stool samples	16S rRNA genes sequencing	NR	NR	ΝP	The gut microbiota profiles of the offspring did not show any correlation with maternal GDM at any age.
Hu et al. [33], 2013, USA	23	10 (4 mothers with pre-gestational type 2 diabetes mellitus)	13	2 h and 48 h after birth	Meconium	16S rRNA sequencing	Phylum *Proteobacteria*	Phylum *Bacteroidetes*. In neonates of mothers with DM: *Bacteroidetes*.	In the meconium samples, taxonomic analyses indicated that the overall bacterial content significantly differed based on maternal diabetes status.
Huang et al., 2021 [38], Thailand	79	I-GDM-S (n = 28) and I-GDM-U (n = 13)	38	NR	Meconium and the first feces	16S rRNA gene sequencing	NR	*Firmicutes*-to-*Bacteroidetes* F:B ratio	*Firmicutes*-to-*Bacteroidetes* F:B ratio was observed significantly higher in both meconium and the first feces of the I-GDM vs. I- non GDM group, and I-GDM-U vs. I-GDM-S group
Huang et al. [35], 2022, Thailand	71	38	33	Within 24 and 48 h after birth	Meconium and first feces	16S rRNA gene sequencing		*Clostridiales*, *Lactobacillus* and *Bacteroidetes* in the GDM-I group *Firmicutes*-to-*Bacteroidetes* F:B ratio	Insulin therapy altered the composition of maternal gut microbiota, potentially influencing the microbiota transferred to the mothers’ newborns.
Li et al. [36], 2023, China	130	74	56	At 5, 42, and 90 days of life	Fecal samples	16S rRNA sequencing, taxonomic assignment, and diversity analysis.	*L. salivarius*, *B. dentium*, and *B. breve*	*L. gasseri* and *B. pseudocatenulatum*	A significant delay in the colonization of Lactobacillus and Bifidobacterium spp. in breast-fed infants born to mothers with GDM was observed, resulting in a distinct gut microbial structure and metabolome.
Ponzo, V. et al. [37], 2019, Italy	48	29	19	3–5 day of life	Fecal samples (following meconium expulsion)	16S rRNA sequencing	Genus: *Staphylococcus*, *Ralstonia*, *Lactobacillus*, and some members of *Enterobacteriaceae*	Phylum *Actinobacteria* and *Bacteroidetes*; Genus: *Escherichia* and *Parabacteroides*	Infants born to mothers with GDM exhibited a higher relative abundance of proinflammatory taxa compared to infants born to healthy women.
Sililas et al. [38]. 2021, Thailand	88	49	39	At 24 and 48 h of life	Meconium	16S rRNA gene sequencing	NR	NR	There was no difference in neonatal gut microbiota between the groups.
Soderborg et al. [39], 2020, USA	46	13	33	At the age of 2 weeks	Fecal samples	16S rRNA gene amplicon sequencing	Gut taxa: *Lactobacillus*, *Flavonifractor*, *Lactobacillaceae*, *Rikenellaceae*, *Erysipelotrichaceae*, and unspecified families in *Gammaproteobacteria*	*Phascolarctobacterium*	GDM, both alone and in conjunction with maternal overweight or obesity, uniquely affects the initial colonization of specific infant microbiota. These patterns may predispose infants to an increased risk of inflammatory and metabolic diseases in the future.
Song et al. [41], 2022, China	28 neonates born to 15 normal pregnant women (NG_NO group), 6 pregnant women with GDM alone (G_NO group), and 7 pregnant women with overweight alone (NG_O group)	6	22	First hours of life	Meconium	16s rRNA double-ended sequenced and bioinformatically analyzed	*f_Nocardioidaceae*	Genus *Diaphorobacter*	The gut microbiota of pregnant women and their newborns is closely associated with obesity and GDM.
Song et al. [40], 2023, China	73	34	39	1 month of age (“M1 phase”) and at 6 months of age (“M6 phase”)	Fecal samples	16S rRNA gene sequencing	*Proteobacteria* phylum, family *Enterobacteriaceae* and its parent order *Enterobacterales* within class *Gammaproteobacteria*	*Actinobacteria* phylum (genus *Bifidobacterium* and its parent taxa] from family to class [*Bifidobacteriaceae*, *Bifidobacteriales*, and *Actinomycetia*] and family *Coriobacteriaceae* and its parent order *Coriobacteriales* within class *Coriobacteriia*	Maternal GDM was associated not only with the community structure and composition of the offspring’s gut microbiota at specific time points but also with differential changes from birth to infancy.
Su et al. [42], 2018, China	34	20 [15 neonates born to mothers with GDM treated by diet and exercise (GDM_A); 1 and 5 neonates born to mothers with GDM treated by insulin in combination with diet and exercise (GDM_A2)])	14	Within 24 h of life	Meconium	16S rRNA gene amplicon sequencing	Phylum *Bacteroidetes*, genus *Prevotella* and *Lactobacillus* within the phylum *Firmicutes*, in GDM_A1 group. *Firmicutes*, *Synergistetes*, *Thermi*, *Spirochaetes*, *Chloroflexi*, and *uryarchaeota* in the GDM group	*Proteobacteria*	Sequencing and bioinformatics analysis of 16S rRNA revealed that the gut microbiota of newborns with GDM differed from that of control newborns. Taxonomy analyses indicated that the overall bacterial composition varied significantly based on maternal diabetes status, with the microbiome of the GDM group exhibiting lower alpha diversity compared to the control group.
Valdez-Palomares et al. [43], 2024 Mexico	40	14	26	0–6 months, 7–12 months, and 13–30 months	Fecal samples	16S rRNA gene QIIME2 and Picrust2	Phylum *Firmicutes*, specifically in *Veillonella* genus	Phylum *Bacteroidetes*, specifically in *Bacteroides*.	The offspring of mothers with GDM exhibit a distinct taxonomic profile characterized by taxa linked to gut microbiota immaturity.
Wang et al., 2018 [44], China	140 neonates (83 samples)	NR	NR	Within 24 h of life	Meconium	16S rRNA gene and metagenomic sequencing	*Faecalibacterium*/ *Fusobacterium* ratios	*Blautia*, *Coprococcus*, *Roseburia*, and *Sutterella* *Lactobacillus*	Microbiota of neonates whose mothers suffered from GDM differs significantly from that of controls.
Zhu et al. [6], 2022, China	120	60	60	Few hours after birth	Meconium	16S rRNA genes sequencing	*Proteobacteria*, at genus level: *Enhydrobacter*, *Psychrobacter*, *Aerococcus*, *Faecalibacterium*, *Herbaspirillum*, *Pelomonas Burkholderia-Caballeronia-Paraburkholderia*, and untitled genus in the family *Enterobacteriaceae*	Genus: *Xanthobacter*, *Cytophaga*, *Serratia*, and *Actinomyces*	The dysbiosis of the gut microbiome induced by maternal GDM may potentially play a significant role in the increased infant BMI during the first 12 months of life.

Abbreviations: 16S ribosomal RNA, rRNA; birth weight, BW; gestational diabetes mellitus, GDM; infants born to mothers with GDM, I-GDM; infants born to mothers with GDM with unsuccessful diet control. I-GDM-U; infants born to mothers with GDM with successful diet control, I-GDM-S; infants from non-GDM mothers, I-non GDM; not reported, NR; oral glucose tolerance test, OGTT.

**Table 3:** Studies evaluating the correlation of GDM with the dynamic changes in gut microbiota in offspring over time.

Author, Year, Location	Study Design	Population (n)	I-GDM	I-Non GDM	Timing of Microbiome Analysis	Sample Type	Study Results
Crusell et al. [2], 2020, Denmark	Cohort	125	43	82	The first week of life and at an average age of 9 months	Fecal samples	Offspring of mothers with GDM initially show lower gut microbiota richness at birth, but by 9 months of age, they catch up and exhibit similar gut bacterial richness as offspring born to normoglycemic mothers.
Li et al. [36], 2023, China	Observational	130	74	56	At 5, 42, and 90 days of life	Fecal samples	A significant delay in the colonization of *Lactobacillus* and *Bifidobacterium* spp. in breast-fed infants born to mothers with GDM was observed, resulting in a distinct gut microbial structure and metabolome.
Song et al. [40], 2023, China	Longitudinal	73	34	39	1 month of age (“M1 phase”) and at 6 months of age (“M6 phase”)	Fecal samples	Maternal GDM was associated not only with the community structure and composition of the offspring’s gut microbiota at specific time points but also with differential changes from birth to infancy.
Valdez-Palomares et al. [43], 2024 Mexico	Cross-sectional	40	14	26	0–6 months, 7–12 months, and 13–30 months	Fecal samples	The offspring of mothers with GDM exhibit a distinct taxonomic profile characterized by taxa linked to gut microbiota immaturity.

Abbreviations: gestational diabetes mellitus, GDM; infants born to mothers with GDM, I-GDM.

## Data Availability

Data are contained within the article.

## Supplementary Materials

The following supporting information can be downloaded at https://www.mdpi.com/article/10.3390/microorganisms12081564/s1, PRISMA checklist.
